# Optical Properties and Growth of the (100) Crystal-Faced MAPbBr_3_ Film on TiN-Buffered MgO Substrate

**DOI:** 10.3390/ma19061265

**Published:** 2026-03-23

**Authors:** Tzu-Lung Chang, Yu-Chen Lin, Yu-Li Hsieh, Hsueh-Hsing Hung, Hui-Huang Hsieh

**Affiliations:** 1 School of Defense Science, Chung Cheng Institute of Technology, National Defense University, 75, Shiyuan Rd., Daxi Dist., Taoyuan 335009, Taiwan; 2 Department of Electrical and Electronic Engineering, Chung Cheng Institute of Technology, National Defense University, 75, Shiyuan Rd., Daxi Dist., Taoyuan 335009, Taiwan; sim20022389@gmail.com (Y.-C.L.); s943208@gmail.com (Y.-L.H.); 3 Department of Physics, National Tsing Hua University, 101, Section 2, Kuang-Fu Road, Hsinchu 300013, Taiwan

**Keywords:** MAPbBr_3_, inverse temperature crystallization, MgO, TiN

## Abstract

The growth of MAPbBr_3_ crystal-faced films is a critical challenge for advancing optoelectronic devices. This study presents a methodology for fabricating a (100) crystal-faced MAPbBr_3_ film on a lattice-matched MgO/TiN composite substrate using a localized thermally driven inverse temperature crystallization technique. The metallic TiN buffer layer offers a 0.66% lattice mismatch to MAPbBr_3_, minimizing interfacial strain. Furthermore, the orientation enhancement of the film induced by post-annealing was confirmed by X-ray diffraction, with the (200) peak FWHM decreasing from 0.042° to 0.028° and the intensity ratio of the (100) to (110) peaks increasing from 6.89 to 19.00. These structural improvements directly translate into enhanced optical performance. The annealed sample exhibited sharper Raman phonon modes at 49 cm^−1^ and 151 cm^−1^, a 1.8-fold photoluminescence intensity enhancement, and a 20.8% narrowing of the PL FWHM at 536 nm. Additionally, UV-Vis spectroscopy confirms the bandgap of MAPbBr_3_, displaying a steeper absorption edge with a bandgap of 2.30 eV. These metrics provide compelling evidence of suppressed non-radiative recombination and improved optical homogeneity after annealing. By integrating TiN as an electron-transport and buffer layer to reduce strain and lattice mismatch, the MgO/TiN/MAPbBr_3_ architecture offers a scalable, scientifically grounded pathway to improve MAPbBr_3_’s optical performance.

## 1. Introduction

In recent years, metal halide perovskites have emerged as promising materials for optoelectronics, particularly in solar cells and photodetectors. Their exceptional optoelectronic properties, including high absorption coefficients, long carrier diffusion lengths, tunable bandgap, and low-cost solution processing, have driven intensive research into scalable, efficient, and stable device architectures [1,2,3]. As perovskite-based devices continue to evolve, thin-film technology has become a critical component in realizing their full potential.

To fabricate high-performance perovskite films, various thin-film deposition techniques have been developed and optimized. Currently, standard deposition methods include spin coating [4], blade coating [5], thermal evaporation [6], and chemical vapor deposition (CVD) [7]. Each technique offers distinct advantages and challenges in terms of scalability, uniformity, film crystallinity, and device integration.

For instance, spin coating is widely used in laboratory-scale research due to its simplicity and ability to produce smooth, controlled-thickness films. However, this method suffers from limited scalability, non-crystal oriented, and material waste. Blade and slot-die coating are better suited to large-area fabrication and roll-to-roll processing, but require precise control of rheological properties and drying kinetics to achieve uniformity. Thermal evaporation and CVD techniques can yield highly uniform films with fewer impurities; however, they typically result in polycrystalline films for ABX_3_ (A = CH_3_NH_3_^+^, CH(NH_2_)_2_^+^, Cs^+^; B = Pb^2+^ or Sn^2+^; X = I^−^, Br^−^, or Cl^−^) perovskite grown on glass, ITO, and silicon substrates.

A significant concern in thin-film perovskite fabrication—especially when using solution-based methods—is the formation of polycrystalline films. While easier to process, polycrystalline perovskite films often exhibit significant drawbacks, including grain boundaries, pinholes, and phase impurities. These structural imperfections act as non-radiative recombination centers, leading to degraded charge carrier transport, lower power conversion efficiencies, and reduced long-term stability [8,9,10]. Moreover, moisture and ion migration along grain boundaries further accelerate material degradation, posing substantial challenges for device reliability. Given these considerations, advancing deposition techniques that promote film quality with low defects remains a vital goal in perovskite device engineering. Compared to iodide-based perovskites such as MAPbI_3_, methylammonium lead bromide (MAPbBr_3_) has demonstrated superior intrinsic stability under ambient conditions. Replacing iodide ions with bromide ions results in a more robust crystal structure due to stronger Pb–Br bonding and a wider bandgap (~2.2 eV), thereby reducing susceptibility to light-induced degradation. Studies have shown that MAPbBr_3_ films retain their crystalline phase and optical properties better than their iodide counterparts during prolonged exposure to heat, light, and oxygen [11,12]. This enhanced thermal and photostability makes MAPbBr_3_ a favorable candidate for applications in stable photodetectors and tandem solar cells where long-term operational durability is crucial.

In addition to its improved thermal and photostability, MAPbBr_3_ also exhibits greater resistance to moisture-induced degradation, a key failure mode in halide perovskite devices. The smaller ionic radius and higher lattice energy of Br^−^ ions result in a more compact and less hygroscopic lattice, thereby reducing water penetration and hydrolysis rates [12]. Structurally, MAPbBr_3_ forms highly oriented thin films with lower defect densities and smoother surfaces, which further limits pathways for moisture ingress. These advantages not only enhance the film’s environmental resilience but also contribute to more consistent and reproducible device performance across fabrication batches. As a result, MAPbBr_3_ has become a superior candidate for scalable, stable thin-film optoelectronic devices operating beyond the laboratory scale.

**Figure 1:**
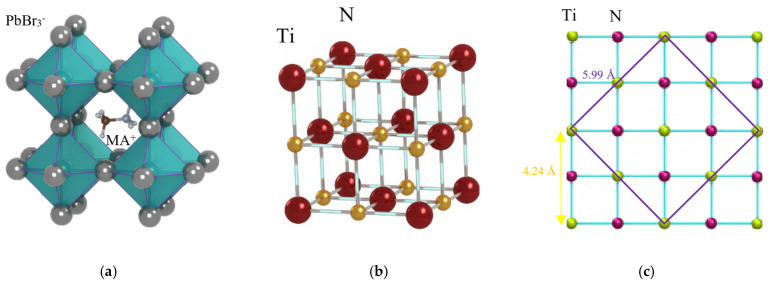
(**a**) MAPbBr_3_ cubic crystal structure, lattice constant 5.93 Å. (**b**) TiN cubic crystal structure, lattice constant 4.24 Å. (**c**) Atoms on top view of TiN (100) crystal plane, the purple line represents the diagonal line of the TiN unit cell.

Lattice matching plays a critical role in the highly oriented growth and crystalline quality of perovskite thin films. For MAPbBr_3_, which has a cubic crystal structure (Figure 1a) with a lattice constant of approximately 5.93 Å [13,14], achieving a lattice-matched substrate can significantly improve film quality by minimizing interfacial strain and promoting oriented crystal growth. This work demonstrates a method for growing crystal-faced MAPbBr_3_ thin film on a lattice-matched substrate, with a conductive layer serving as an electrode. The cubic MgO was chosen as the base substrate, and a metallic TiN film was used as the buffer layer. Figure 1b shows the cubic TiN crystal structure; the lattice mismatch is approximately 0.67% between them, as the lattice constants of MgO and TiN are 4.212 Å and 4.241 Å (JCPDS file no. 38-1420), respectively [15,16,17,18,19]. Figure 1c illustrates the lattice matching mechanism between TiN and MAPbBr_3_, facilitated by the diagonal length of the TiN primitive unit cell, which is 5.998 Å (√2 × 4.241 Å), closely matching the lattice constant of 5.93 Å of MAPbBr_3_. The diagonal line also defines the TiN unit cell, which includes two primitive unit cells. The close lattice matching between them enables coherent oriented growth, minimizing interface dislocations and providing a structurally ideal platform for the development of a MAPbBr_3_ film.

For MAPbBr_3_ with valence band maximum (VBM) −5.7 eV and conduction band minimum (CBM) −3.4 eV [20,21], the metallic TiN layer with a work function of 4.7 eV can act as an electron transport layer (ETL) [22,23]. TiN not only offers efficient electron extraction but also serves as a chemical barrier, protecting the interface from moisture and halide-induced corrosion. Crystal structure and optical characterization of MgO/TiN/MAPbBr_3_ in this work were carried out by X-ray diffraction (XRD), Field emission scanning electron microscopy (FESEM), Raman spectroscopy, photoluminescence (PL), and UV-Vis. The experimental results affirm the critical role of substrate lattice matching for highly oriented MAPbBr_3_ growth and optical performance.

## 2. Materials and Methods

### 2.1. TiN Buffer Layer Deposition

Metallic TiN films typically serve as electrode materials. Here, the epitaxial TiN film serves as a buffer layer to alleviate strain at the interface between the substrate and the MAPbBr_3_ film. TiN films were deposited on a MgO (100) single-crystal substrate using unipolar reactive-pulse DC magnetron sputtering with a Ti target and nitrogen gas. Before deposition, MgO (100) substrates were cleaned using the standard RCA process to remove organic and particulate contaminants. This involved sequential ultrasonic cleaning in acetone, isopropanol (IPA), and deionized (DI) water for 10 min. Next, the substrates were dried with nitrogen (N_2_) gas to eliminate residual moisture. After the cleaning step, a 129 nm titanium nitride (TiN) layer was deposited via reactive pulse DC magnetron sputtering. Before deposition, the chamber was evacuated to a base pressure of approximately 7 × 10^−7^ mTorr to ensure a clean vacuum environment. Throughout the sputtering process, the chamber pressure was maintained at 1 mTorr, with a nitrogen flow rate of 3 sccm. The power, frequency, and reverse time of the pulse DC sputter parameters were 660 W, 50 kHz, and 1.0 μs, respectively. The Ti target surface was pre-sputtered for 2 min to remove surface oxides and contaminants before film deposition. The TiN deposition time, substrate temperature, and substrate bias voltage were set to 30 min, 350 °C, and −60 V, respectively. The metallic nature of the deposited TiN film was confirmed by a resistivity of 190 µΩ·cm measured with a Keithley 2400 multimeter (Tektronix, Beaverton, OR, USA).

### 2.2. Growth of MAPbBr_3_ on MgO/TiN

Lead (II) bromide (PbBr_2_ 99.99%, MW = 367.01 g/mol), methylammonium bromide (MABr > 99.5%, MW = 111.97 g/mol) powders, and DMF (Dimethylformamide) solvents were acquired from Ruilong Optoelectronics Corp., Luminescence Technology Corp., and Sigma-Aldrich, respectively. The purity of all received materials was verified using Raman spectroscopy and was used without further purification. To prepare the precursor solution, equimolar amounts of methylammonium bromide (MABr) and lead bromide (PbBr_2_) were dissolved in N,N-dimethylformamide (DMF). The PbBr_2_ and MABr powders were mixed in a cooled DMF solvent under magnetic stirring overnight to ensure complete dissolution of the solutes. The precursor solution was filtered through a 0.2 μm PTFE filter to remove particulates and undissolved residues.

**Figure 2:**
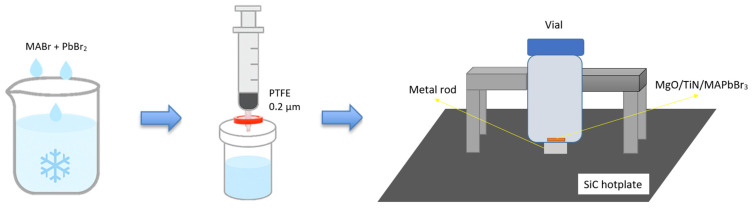
Schematic of the experimental setup for MAPbBr_3_ growth on MgO/TiN substrate.

After filtration, the precursor solution was placed on a temperature-controlled silicon carbide (SiC) heating platform, and the MgO/TiN substrate was positioned in the central heating zone at the bottom of the vial. The growth of MAPbBr_3_ on the substrate was achieved using the localized thermally driven inverse temperature crystallization (LTDITC) method. The schematic diagram is shown in Figure 2; details were reported in our previous study [24]. The heating rate of the hotplate was 0.02 °C/min from 60 to 90 °C. The thermal gradient between the inner and outer zones of the heating metal rod was controlled. The inner zone was maintained at a saturation temperature (between the undersaturated and oversaturated curves) to promote deposition. In contrast, the outer zone was kept below the undersaturated point to prevent spontaneous nucleation. Rapid thermal annealing (RTA) at 325 °C for 25 s was performed to enhance the crystallization of MAPbBr_3_ grains on the substrate.

The rapid thermal annealing temperature of 325 °C we used was derived from experimental results with different annealing times and temperatures. We found that the benefit of lattice reorganization and residual solvent removal is greater than that of partial decomposition by RTA at 325 °C for 25 s. In our experiment, the annealing time during our rapid thermal annealing (RTA) is strictly limited to 25 s.

### 2.3. Characterization of MAPbBr_3_ Samples

The structural characteristics of MAPbBr_3_ thin films were investigated through X-ray diffraction (XRD) and Raman spectroscopy. XRD measurements were conducted at Temporally Coherent X-ray Diffraction beamline 09A of Taiwan Photon Source in the National Synchrotron Radiation Research Center (NSRRC), Taiwan. The microscope images were taken by Hitachi FESEM SU8600B (Tokyo, Japan) and JEOL JSM-IT770HR (Tokyo, Japan) with 10 kV electron accelerating voltage. To detect molecular vibration modes, Raman spectra were collected at room temperature using a HORIBA iHR-550 (Piscataway, NJ, USA) confocal modular Raman microscope equipped with a 785 nm excitation laser. To evaluate the optical properties of MAPbBr_3_, photoluminescence (PL) spectroscopy was performed at room temperature using a 325 nm He-Cd laser as the excitation source. All Raman and PL measurements were conducted using a high-precision XYZ movable translation sample stage to position the measurement exactly at the center of the samples before and after annealing, and a movable *z*-axis to maintain a consistent focal plane and minimize incident beam intensity variation since most intensity differences in measurements of different samples came from the misalignment of focus on the focal plane. To accurately probe low-frequency modes (such as the TO mode at 49 cm^−1^), we used a 785 nm laser equipped with a laser line filter. A double infrared 785 nm edge filter is installed at the front of the manochromometer to filter the reflected laser light from the sample, and a 1200/mm grating is used to effectively capture these low-wavenumber signals. Furthermore, a 50× objective lens was employed to collect Raman fluorescence from the thin films, ensuring a good signal-to-noise (S/N) ratio.

Additionally, UV-Vis absorbance spectra were acquired in transmittance mode using a UH4150 spectrophotometer, enabling determination of the material’s optical bandgap and assessment of the film’s absorption behavior.

## 3. Results and Discussion

### 3.1. X-Ray Diffraction Analysis

**Figure 3:**
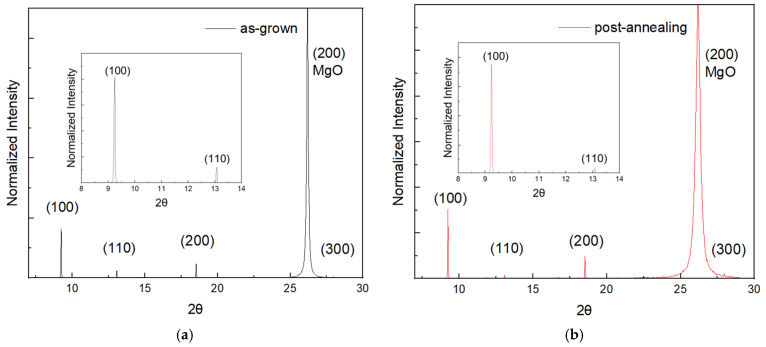

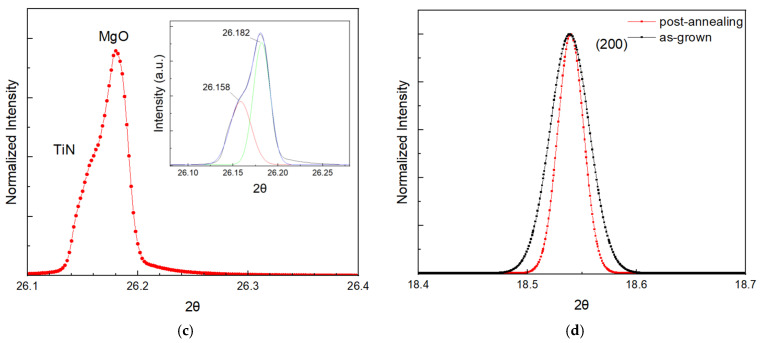
XRD patterns of MAPbBr_3_ deposited on MgO/TiN, insets are enlarged pictures (**a**) as-grown. (**b**) post-annealing. (**c**) high-resolution peaks of MgO (200) (green line)/TiN (200) (red line) and their fitting (inset). (**d**) high resolution (200) peaks of MAPbBr_3_.

Figure 3 shows the XRD patterns of LTDITC MAPbBr_3_ thin films on a MgO/TiN substrate, which was selected due to its well-matched lattice constant and its role as an electron transport layer (ETL). The energy of the monochromatic X-ray source was 12.985 keV (λ = 0.95479 Å). The diffraction profile in Figure 3a shows the XRD result of the as-grown film. Unlike polycrystalline films grown by traditional methods—such as single-step spin coating, the two-step process (PbBr_2_ spin coating followed by MABr dipping), chemical vapour deposition, or physical evaporation—which show non-face-oriented multiple diffraction peaks from crystal planes (100), (110), (200), (210), (211), (220), (300), (310), (311), (320), and (321) [7,25,26,27], the XRD of films grown by the LTDITC method exhibit firm (100), (200), and (300) and a minor (110) diffraction peaks, indicating a highly face oriented film formed on MgO/TiN substrate. To further enhance the crystal quality, thermal post-annealing was applied.

The (100) and (110) planes diffraction peaks are shown in the inset of Figure 3a,b. The (100) to (110) peak intensity ratio increased from 6.89 to 19.00 after post-annealing of the sample, indicating a significant enhancement in crystal quality of the (100) plane induced by thermal annealing. Such improvement suggests that long-range ordering of (100)-oriented crystal grains on the substrate was enhanced by annealing, thereby contributing to the suppression of defects in the film.

The high-resolution MgO (200)/TiN (200) peaks are shown in Figure 3c, and the inset shows the peak fit. The fitting peaks at 26.158° and 26.182° are attributed to deposited TiN (200) and substrate MgO (200) crystal planes, respectively [15,16,17,18,19]. The calculated TiN and MgO lattice constants are 4.219 and 4.215 Å from the values of fitting peaks, respectively. The lattice mismatch between them was reduced to 0.09%, while the theoretical lattice mismatch is 0.67%. The 0.022 Å lattice constant reduction in deposited TiN in comparison with the standard TiN lattice constant of 4.241 Å of powder (JCPDS file no. 38-1420) induces compressive stress at the interface between the deposited TiN layer and MgO substrate. The diagonal length of the TiN buffer layer in Figure 1c was reduced from 5.998 Å to 5.966 Å, which is very close to the lattice constant of 5.93 Å of the MAPbBr_3_ crystal.

Figure 3d presents the high-resolution (200) XRD peaks of the MAPbBr_3_ thin films. The lattice constants of both MAPbBr_3_ films were 5.927 Å, calculated from the (200) peak position at 18.539°. The lattice constant is consistent with the value reported for single crystals (5.93 Å) [13,14]. The result contrasts with the lattice constant deviations of thin films from single-crystal values reported in the literature, which are attributed to lattice strain [13,28,29,30,31]. Reported lattice constants range from 6.08 Å (tensile strain) to 5.90 Å (compressive stress). The minor lattice-mismatch-induced deviation in the MAPbBr_3_ film on the MgO/TiN substrate in this work relaxes the interface strain. The diagonal length of the unit cell in the deposited TiN film is 5.967 Å, which provides a near-perfect geometric arrangement with a lattice mismatch of 0.66% relative to the MAPbBr_3_ lattice (5.927 Å). This configuration effectively mitigates the epitaxial strain that typically distorts films grown on lattice-mismatched substrates. Furthermore, the FWHM of the post-annealed sample (0.028°) is significantly lower than that of the as-grown film (0.042°), indicating that crystalline quality improved with post-annealing.

### 3.2. Field Emission Scanning Electron Microscopy Analysis

**Figure 4:**
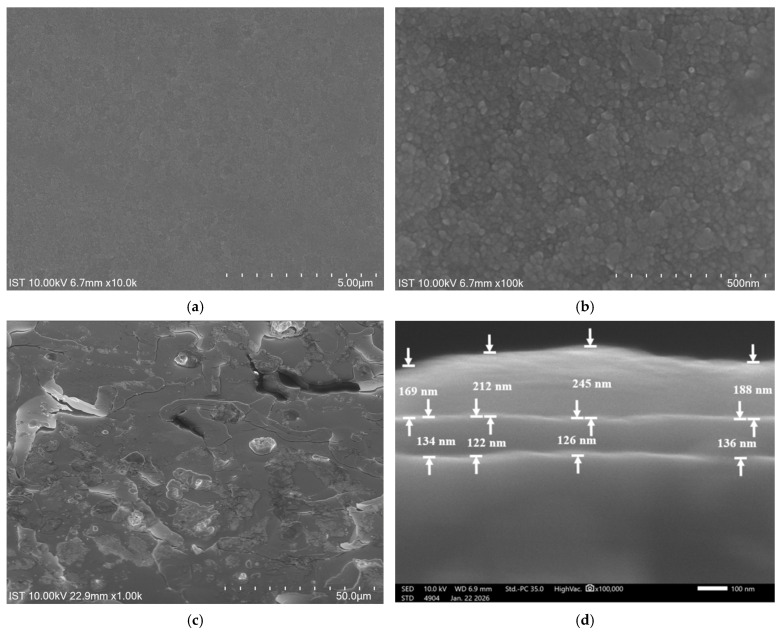
SEM images of MgO/TiN top view (**a**) at 10 k magnification, (**b**) at 100 k magnification, and SEM images of MgO/TiN/MAPbBr_3_ after annealing, (**c**) top view at 1 k magnification, and (**d**) cross section of one micro planar sheet at 100 k magnification.

The surface morphology of the TiN thin film deposited onto a MgO substrate is shown in Figure 4a,b. The TiN film surface is smooth and free of pinholes. Figure 4b shows that the average grain size is 24 nm in a 100k-magnification SEM image of the film. Figure 4c shows the surface morphology of the MAPbBr_3_ grown on the MgO/TiN composite substrate after annealing, viewed at a 45° tilt. The deposited MAPbBr_3_ formed a predominantly planar sheet, with minor surface grains and pores. The thicknesses of the TiN and MAPbBr_3_ layers are shown in Figure 4d. The average thickness of the TiN buffer layer is 129 nm (range from 122 to 136 nm), while the average thickness of the MAPbBr_3_ micro sheet is 203 nm (range from 169 nm to 245 nm).

### 3.3. Raman Spectroscopy Analysis

**Figure 5:**
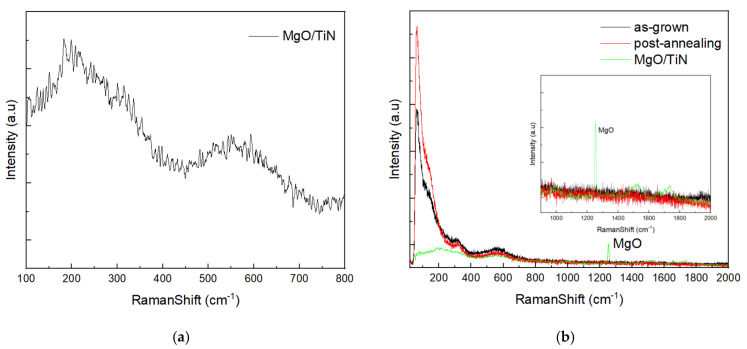
Raman spectra of (**a**) MgO/TiN thin film and (**b**) MgO/TiN/MAPbBr_3_ as-grown (black), and post-annealing (red), and MgO/TiN thin film (green).

In Figure 5a, the TiN Raman spectrum exhibits a broad transverse acoustic (TA) mode peak at 220 cm^−1^ and a longitudinal optical (LO) mode peak at 550 cm^−1^ [32]. The Raman curves of MgO/TiN and MgO/TiN/MAPbBr_3_ films are shown in Figure 5b, and the inset shows the region above 900 cm^−1^. The Raman shifts from the underlying MgO/TiN substrate, characterized by broad bands at 220 cm^−1^ and 550 cm^−1^, are attributed to TiN, and a distinct peak at 1253 cm^−1^ is attributed to MgO. While these substrate signatures are discernible in the as-grown samples, they are mitigated after post-annealing. Furthermore, the key Raman-active vibrational modes associated with the MAPbBr_3_ perovskite structure are distinguishable from the background signal of MgO/TiN. Table 1 lists the reported characteristic Raman shifts of MAPbBr_3_ [33]. In the Raman spectra of our samples, the Raman shift peak at 49 cm^−1^ corresponds to a transverse optic (TO) mode related to octahedral twisting. In comparison, the peak at 151 cm^−1^ is attributed to the MA^+^ librational mode, involving vibration around the central C–N axis of the methylammonium (MA^+^) cation.

After post-annealing, both Raman shift peaks of MAPbBr_3_ at 49 cm^−1^ and 151 cm^−1^ become sharper and more intense, indicating improved crystallinity and reduced dynamic disorder within the crystal lattice. Specifically, the 49 cm^−1^ peak intensity increased by a factor of 1.32, while the 151 cm^−1^ peak shows a 1.47-fold enhancement, indicating improved crystallinity and fewer defects. These improvements support the claim that thermal annealing facilitates the reordering of molecules and the better alignment of organic cations within the structure. The Raman results align well with the XRD findings, providing additional evidence of the beneficial effects of annealing on the structural and optoelectronic properties of MAPbBr_3_.

### 3.4. Photoluminescence and UV-Vis Absorbance Characterization

**Figure 6:**
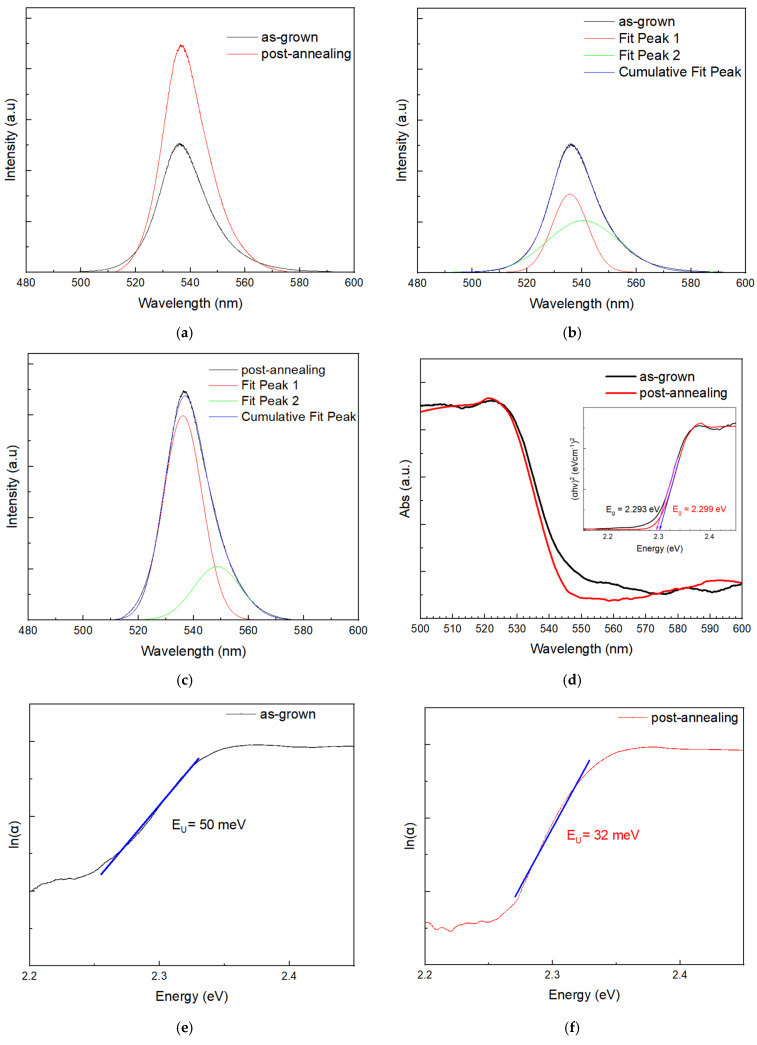
Photoluminescence and UV-Vis spectra of MgO/TiN/MAPbBr_3_. (**a**) before and after thermal annealing. (**b**) The fitting spectrum before annealing. (**c**) The fitting spectrum after annealing. (**d**) Normalized UV-Vis absorbance and Tauc plot (inset) for as-grown and post-annealed samples. Co-relationship of absorption coefficient (α) and photon energy for the Urbach energy calculation (**e**) as-grown and (**f**) post-annealed samples.

Figure 6a shows the room-temperature photoluminescence spectra of MAPbBr_3_ thin films before and after thermal annealing. Both spectra exhibit a strong emission peak centered near 536 nm, characteristic of MAPbBr_3_ [34,35]. The PL intensity of the sample increased by a factor of 1.8 after post-annealing. This substantial improvement in emission intensity indicates reduced nonradiative recombination, driven by enhanced crystallinity and improved grain boundary passivation. Additionally, the annealed film exhibits a narrower FWHM, decreasing from 24 nm (pre-annealed) to 19 nm (post-annealed), suggesting a more uniform electronic structure and lower defect density.

Figure 6b,c is fitting curves of peaks in Figure 6a using Gaussian function. The resulting components, Peak 1 and Peak 2, are attributed to direct-bandgap recombination and trap-state recombination, respectively [36]. These trap states, which reside energetically beneath the conduction band minimum, typically arise from native crystal defects and surface imperfections [36,37,38]. The intensity ratio of Peak 1 to Peak 2 increased significantly from 1.485 (before annealing) to 3.749 (after annealing), indicating that the defect density of the thin film was effectively reduced by post-annealing.

Figure 6d presents the normalized UV-Vis absorbance spectra of MAPbBr_3_ thin films before and after annealing. Both spectra exhibit a distinct absorption edge at approximately 542 nm. The Tauc plot shows that the bandgap of both samples is nearly identical, as the bandgap of the grown sample is 2.293 eV and the bandgap of the post-annealing sample is 2.299 eV, which is consistent with the reported bandgap of the MAPbBr_3_ film (2.3 eV) [39,40]. After annealing, the absorbance edge exhibits a steeper slope, indicating improved crystallinity and optical uniformity, which are crucial for high-efficiency optoelectronic applications.

Perovskites often contain a significant density of sub-gap states due to crystalline defects that extend just below the conduction band edge, leading to positive-negative charge recombination. That causes the material’s absorption coefficient α to decrease exponentially as the photon energy drops below the bandgap, creating a tail in the UV-Vis absorbance spectrum. This exponential relationship, first described by Urbach, is expressed by Equation (1):

(1) $$\alpha \left(E\right)=\;\alpha_{0}\exp\left(\frac{E-E_{C}}{E_{U}}\right)$$

where α is the absorption coefficient, *α*_0_ and $E_{C}$ are material-dependent constants, E is the incident photon energy, and $E_{U}$ is the Urbach energy, which depends on the slope of the exponential function. The $E_{U}$ is a critical indicator of energetic disorder, while ideal single crystals possess near-zero structural disorder; less crystalline or disordered materials exhibit pronounced Urbach tails [41,42]. Band tails originate from various sources, including crystal lattice defects, electron-phonon coupling, and carrier-impurity interactions. Because these localized tail states extend into the bandgap, they act as trap centers that directly accelerate non-radiative, trap-assisted recombination and degrade device performance. As shown in Figure 6e,f, the extracted Urbach energy decreased significantly from 50 meV for the as-grown film to 32 meV for the post-annealed film. The values are lower than the reported 92 meV for MAPbSnBr3 perovskite crystals obtained via the antisolvent vapor-assisted crystallization method in the literature. This quantitative reduction confirms that the post-annealing process effectively suppresses structural disorder and sub-gap defect states. The results are consistent with the increase in the peak ratio of the main peak to the tail peak in PL.

It is important to note that MAPbBr_3_ undergoes structural phase transitions at different temperatures, whereas the MgO/TiN substrate remains in a stable cubic phase. Because the 0.66% lattice mismatch between the buffered TiN layer and MAPbBr_3_ relies on both materials being in the cubic phase, the optical and structural improvements demonstrated in this study are specifically evaluated at room temperature to exclude the effect of the structure phase transition.

## 4. Conclusions

In this study, the lattice-matched MgO/TiN composite substrate for MAPbBr_3_ was fabricated by pulsed DC sputtering. The lattice mismatch of deposited TiN buffer layer to the MgO substrate was reduced from 0.67% (theoretical value) to 0.09% (experimental value). This lattice convergence optimizes the TiN diagonal length, creating a superior template for the MAPbBr_3_ perovskite. We established a methodology for fabricating (100) crystal-faced MAPbBr_3_ on a lattice-matched MgO/TiN substrate using the LTDITC technique. A key advantage of this architecture is the strain accommodation, as the lattice mismatch was reduced from 1.00% (theoretical value) to 0.66% (experimental value) between the TiN buffer layer and MAPbBr_3_, which can effectively mitigate interfacial strain and lay the foundation for high-quality, oriented thin film growth by various deposition methods.

Furthermore, the crystallinity and crystal orientation of samples fabricated by inverse temperature crystallization were significantly enhanced by post-annealing. This improvement was confirmed by a reduction in the X-ray diffraction FWHM of the (200) peak from 0.042° to 0.028° and an increase in the peak intensity ratio of the (100) to (110) planes from 6.89 to 19.00.

Crucially, these structural enhancements following post-annealing directly lead to superior optical properties. The annealed sample exhibited sharper Raman phonon modes, a 1.8 times photoluminescence intensity enhancement, and 20.8% narrowing of the PL FWHM. These metrics provide compelling evidence of suppressed non-radiative recombination. Additionally, a steeper absorption edge in the UV-Vis spectrum of the MAPbBr_3_ thin film after annealing confirms a bandgap of 2.30 eV and optical homogeneity. The deposited metallic TiN film serves as a multifunctional layer including a lattice template, effective electron transport, and a chemical barrier. This work presents a composite MgO/TiN substrate for growing highly oriented MAPbBr_3_ films and a viable strategy to improve the performance of MAPbBr_3_ perovskite optoelectronic devices.

## Figures and Tables

**Table 1:** Raman shift of MAPbBr_3_ perovskite [33].

Raman Shift (cm^−1^)	Band Assignment
49	Octahedral twist transverse optic (TO)
151	MA^+^ librational mode
321	C-N torsion
913	C-N asymmetric bend
967	C-N stretching

## Data Availability

The original contributions presented in this study are included in the article. Further inquiries can be directed to the corresponding author.

## References

[1] Zhang D., Zhang Q., Zhu Y., Poddar S., Zhang Y., Gu L., Zeng H., Fan Z. (2023). Metal halide perovskite nanowires: Synthesis, integration, properties, and applications in optoelectronics. Adv. Energy Mater..

[2] Wang H., Sun Y., Chen J., Wang F., Han R., Zhang C., Kong J., Li L., Yang J. (2022). A review of perovskite-based photodetectors and their applications. Nanomaterials.

[3] Li X., Aftab S., Mukhtar M. (2025). Exploring nanoscale perovskite materials for next-generation photodetectors: A comprehensive review and future directions. Nano-Micro Lett..

[4] Tan Z.K., Moghaddam R.S., Lai M.L., Docampo P., Higler R., Deschler F., Price M., Sadhanala A., Pazos L.M., Credgington D. (2014). Bright light-emitting diodes based on organometal halide perovskite. Nat. Nanotechnol..

[5] Yang Z., Chueh C.C., Zuo F., Kim J.H., Liang P.W., Jen A.K.Y. (2015). High-performance fully printable perovskite solar cells via blade-coating technique under the ambient condition. Adv. Energy Mater..

[6] Siegrist S., Yang S.C., Gilshtein E., Sun X., Tiwari A.N., Fu F. (2021). Triple-cation perovskite solar cells fabricated by a hybrid PVD/blade coating process using green solvents. J. Mater. Chem. A.

[7] Sadullah M.D., Ghosh K. (2023). Low-temperature deposition of perovskite CH3NH3PbX3 (X= I, Br) thin films using aerosol assisted CVD via vertical flow for photovoltaic applications. ES Energy Environ..

[8] Jariwala S., Sun H., Adhyaksa G.W., Lof A., Muscarella L.A., Ehrler B., Garnett E.C., Ginger D.S. (2019). Local crystal misorientation influences non-radiative recombination in halide perovskites. Joule.

[9] Feng X., Chen R., Nan Z.A., Lv X., Meng R., Cao J., Tang Y. (2019). Perfection of perovskite grain boundary passivation by Eu-porphyrin complex for overall-stable perovskite solar cells. Adv. Sci..

[10] Soopy A.K.K., Liu S., Najar A. (2024). Enhancement of Photodetector Characteristics by Zn-Porphyrin-Passivated MAPbBr_3_ Single Crystals. Nanomaterials.

[11] Chi W., Banerjee S.K. (2021). Stability improvement of perovskite solar cells by compositional and interfacial engineering. Chem. Mater..

[12] Boyd C.C., Cheacharoen R., Leijtens T., McGehee M.D. (2018). Understanding degradation mechanisms and improving stability of perovskite photovoltaics. Chem. Rev..

[13] Faghihnasiri M., Izadifard M., Ghazi M.E. (2020). Study of strain effects on electronic and optical properties of CH3NH3PbX3 (X = Cl, Br, I) perovskites. Phys. B Condens. Matter.

[14] Grover K., Shree Choudhary J., Dhawan P., Jha R., Yadav H. (2024). Structural and optical properties of ambient-grown hybrid perovskite single crystals: A multidimensional analysis into mechanical and photodetection studies. Opt. Mater..

[15] Barad C., Kimmel G., Opalińska A., Gierlotka S., Łojkowski W. (2024). Lattice variation as a function of concentration and grain size in MgO-NiO solid solution system. Heliyon.

[16] Socha R.P., Szczepanik-Ciba M., Powroźnik W., Spiridis N., Korecki J. (2014). Epitaxial α-Mn (001) films on MgO (001). Thin Solid Films.

[17] Mao W., Qi R., Wu J., Zhang Z., Wang Z. (2024). Evolution of chemical, structural, and mechanical properties of titanium nitride films with different thicknesses fabricated using pulsed DC magnetron sputtering. Materials.

[18] Briggs J.A., Naik G.V., Zhao Y., Petach T.A., Sahasrabuddhe K., Goldhaber-Gordon D., Melosh N.A., Dionne J.A. (2017). Temperature-dependent optical properties of titanium nitride. Appl. Phys. Lett..

[19] Xu W., Zhang Q.Y., Zhou N., Peng B., Shen Y. (2024). Epitaxial growth of nano-texturized NiO films on MgO (001) substrates by a reactive magnetron sputtering method. J. Cryst. Growth.

[20] Guo S., Qiao S., Liu J., Ma J., Wang S. (2022). Greatly improved photoresponse in the MAPbBr_3_/Si heterojunction by introducing an ITO layer and optimizing MAPbBr_3_ layer thickness. Opt. Express.

[21] Liang Y., Wang Y., Mu C., Wang S., Wang X., Xu D., Sun L. (2018). Achieving high open-circuit voltages up to 1.57 V in hole-transport-material-free MAPbBr_3_ solar cells with carbon electrodes. Adv. Energy Mater..

[22] Calzolari A., Catellani A. (2020). Controlling the TiN electrode work function at the atomistic level: A first principles investigation. IEEE Access.

[23] Zhuang Y., Liu Y., Xia H., Li Y., Li X., Li T. (2022). Effective work function of TiN films: Profound surface effect and controllable aging process. AIP Adv..

[24] Chang T.L., Hsieh Y.L., Hung H.H., Chiou J.W., Hsieh H.H. (2025). Thermally driven growth of MAPbBr_3_ single crystals. J. Cryst. Growth.

[25] Chen L.C., Lee K.L., Lin S.E. (2018). Observation of hybrid MAPbBr_3_ perovskite bulk crystals grown by repeated crystallizations. Crystals.

[26] Mehdi H., Mhamdi A., Hannachi R., Bouazizi A. (2019). MAPbBr 3 perovskite solar cells via a two-step deposition process. RSC Adv..

[27] Fru J.N., Nombona N., Diale M. (2020). Synthesis and characterisation of methylammonium lead tri-bromide perovskites thin films by sequential physical vapor deposition. Phys. B Condens. Matter.

[28] Poglitsch A., Weber D. (1987). Dynamic disorder in methylammoniumtrihalogenoplumbates (II) observed by millimeter-wave spectroscopy. J. Chem. Phys..

[29] Létoublon A., Paofai S., Ruffle B., Bourges P., Hehlen B., Michel T., Ecolivet C., Durand O., Cordier S., Katan C. (2016). Elastic constants, optical phonons, and molecular relaxations in the high temperature plastic phase of the CH3NH3PbBr3 hybrid perovskite. J. Phys. Chem. Lett..

[30] Egger D.A., Kronik L. (2014). Role of dispersive interactions in determining structural properties of organic–inorganic halide perovskites: Insights from first-principles calculations. J. Phys. Chem. Lett..

[31] Giorgi G., Fujisawa J.I., Segawa H., Yamashita K. (2014). Cation role in structural and electronic properties of 3D organic–inorganic halide perovskites: A DFT analysis. J. Phys. Chem. C.

[32] Saoula N., Djerourou S., Yahiaoui K., Henda K., Kesri R., Erasmus R.M., Comins J.D. (2010). Study of the deposition of Ti/TiN multilayers by magnetron sputtering. Surf. Interface Anal..

[33] Muslimawati R.M., Manawan M., Takahashi K., Furukawa Y., Bahtiar A. (2022). Single crystal perovskite MAPbBr_3_ prepared by using anti-solvent vapor-assisted crystallization method. J. Phys. Conf. Ser..

[34] Li S., Zhang C., Song J.J., Xie X., Meng J.Q., Xu S. (2018). Metal halide perovskite single crystals: From growth process to application. Crystals.

[35] Mahen E.C.S., Nuryadin B.W., Permatasari F.A., Aimon A.H., Suprijadi, Iskandar F. (2021). A Stable Photoluminescence of Waste Derived Acrylic Plastics (PMMA) and MAPbBr 3 Composite Film. J. Phys. Conf. Ser..

[36] Zhang H., Zhang B., Wang X., Shao W., Nie J., Liu J., Ouyang X., Xu Q. (2020). Surface polished of bulk methylammonium lead tribromide single crystal. Curr. Appl. Phys..

[37] Wang Y., Zhu L., Du C. (2021). Polarization-sensitive light sensors based on a bulk perovskite MAPbBr_3_ single crystal. Materials.

[38] Konda S.R., Lin Y., Rajan R.A., Yu W., Li W. (2023). Measurement of optical properties of CH3NH3PbX3 (X= Br, I) single crystals using terahertz time-domain spectroscopy. Materials.

[39] Ferdowsi P., Ochoa-Martinez E., Alonso S.S., Steiner U., Saliba M. (2020). Ultrathin polymeric films for interfacial passivation in wide band-gap perovskite solar cells. Sci. Rep..

[40] Zhang M., Lifang W., Gong S., Han Q., Wu W. (2021). Forming laterally structured heterojunction with FAPbI3 film for improving performance of MAPbBr_3_ photodetectors. Opt. Mater..

[41] Pacio Castillo A., Medina Velazquez D.Y., Pacio Castillo M., Reyes Miranda J., Hernández M., Serrano De la Rosa L.E., Antonio Hernández C., Cortez Santiago A. (2024). Obtaining and characterization of MAPbSnBr3 perovskite crystals trough AVC method. J. Phys. Conf. Ser..

[42] Subedi B., Li C., Chen C., Liu D., Junda M.M., Song Z., Yan Y., Podraza N.J. (2022). Urbach energy and open-circuit voltage deficit for mixed anion–cation perovskite solar cells. ACS Appl. Mater. Interfaces.

